# Effect of *Lactobacillus plantarum* KSFY01 on the exercise capacity of D-galactose-induced oxidative stress-aged mice

**DOI:** 10.3389/fmicb.2022.1030833

**Published:** 2022-12-22

**Authors:** Qiuping Chen, Chuannan Liu, Yinglong Zhang, Shuai Wang, Fang Li

**Affiliations:** ^1^Department of Education Management, Our Lady of Fatima University, Valenzuela, Philippines; ^2^School of Physical Education and Sports Science, South China Normal University, Guangzhou, China; ^3^School of Physical Education, Yan’an University, Yan’an, China; ^4^The First Middle School of Tongliao City, Tongliao, China; ^5^Collaborative Innovation Center for Child Nutrition and Health Development, Chongqing Engineering Research Center of Functional Food, Chongqing Engineering Laboratory for Research and Development of Functional Food Chongqing University of Education, Chongqing, China

**Keywords:** *Lactobacillus plantarum*, D-galactose, oxidative, exercise, mRNA

## Abstract

**Objectives:**

Aging is a process that involves comprehensive physiological changes throughout the body, and improvements in the exercise capacity of individuals may delay aging and relieve fatigue. Probiotics are subject to ongoing research to investigate their antioxidant properties. The purpose of this study was to investigate the effect of the probiotic *Lactobacillus plantarum* KSFY01 (*L. plantarum* KSFY01) on exercise tolerance in mice induced into a state of accelerated physiological aging by oxidative stress.

**Methods:**

A mouse model of accelerated aging was established using D-galactose to induce oxidative stress. The bacteria *L. plantarum* KSFY01 was isolated from fermented yak yogurt. The effect of *L. plantarum* KSFY01 on the improvement of exercise capacity in aging-accelerated mice was evaluated by measuring their running time until exhaustion, histopathological sections, related biochemical indicators, and underlying gene expression.

**Results:**

The oral administration of *L. plantarum* KSFY01 prolonged the running time of mice and reduced their creatine kinase (CK), alanine aminotransferase (ALT), and aspartate aminotransferasem (AST) levels. From this study, we observed that *L. plantarum* KSFY01 significantly improved the exercise capacity of mice and alleviated liver damage. Treatment with *L. plantarum* KSFY01 reduced the blood urea nitrogen (BUN), lactic acid (LD) accumulation, and lactate dehydrogenase (LDH) elevations produced by the accelerated aging state, and also reversed the changes in muscle glycogen (MG). Overall, *L. plantarum* KSFY01 could effectively improve metabolite accumulation, thereby relieving fatigue in exercised mice. The results of the antioxidant indices *in vivo* showed that *L. plantarum* KSFY01 intervention increased the activity of antioxidant enzymes, decreased the level of malondialdehyde (MDA), and restored the balance between the oxidative and antioxidant systems in fatigued mice. By investigating the underlying molecular mechanism, our results showed that *L. plantarum* KSFY01 intervention significantly reversed the decline in the expression levels of nuclear factor-erythroid 2 related factor 2 (Nrf2) signaling pathway-related factors and improved the body’s antioxidant capacity. We determined that the underlying molecular mechanism responsible for the antioxidant effect of *L. plantarum* KSFY01 mainly involves the activation of the Nrf2 pathway. The effect of *L. plantarum* KSFY01 was dose-dependent, and the expression level of Nrf2 increased with increasing dosage of the probiotic.

**Conclusion:**

This study demonstrated that the probiotic *L. plantarum* KSFY01 exerts antioxidant effects and improved the athletic ability of mice. These findings are of significance to the development and utilization of probiotic resources.

## 1. Introduction

Aging is a process that involves comprehensive physiological changes throughout the body, and is one that all biological individuals must experience (Wang W. et al., 2022; Wang L. et al., 2022). At present, the aging population is expanding, and it is estimated that the global population over the age of 60 will reach 2.1 billion by 2050 (Leeson, 2018). Aging reduces the metabolic rate, imbalances the antioxidant system, and results in an excess of free radicals due to their reduced clearance, thereby resulting in their accumulation in the body (Adetuyi et al., 2022). Simultaneously, the content of lipid peroxide increases in the plasma and tissues, and the anti-fatigue ability of the body also gradually decreases (Zheng et al., 2017). Studies have found that telomere length is preserved in healthy elderly people who practise vigorous aerobic exercise, being positively correlated with the maximum aerobic exercise capacity, and thereby indicating that a high capacity for exercise may contribute to delaying the process of aging (LaRocca et al., 2010). People who regularly exercise have slower heart rates, lower blood pressure, and lower cholesterol levels (Myers et al., 2007). These indicators directly reflect the association between exercise capacity and aging. The decline in organ and muscular function as well as the increase in fatigue caused by aging all contribute to the decline of exercise capacity. To counter this, maintaining an exercise regime can help improve bodily functions, alleviate fatigue, and maintain the overall vitality of the body. The process of aging interacts with the exercise capacity of an individual, whereby bodily health is not only the embodiment of a high capacity for exercise, but can also promote continuous exercise in a positive feedback loop, so as to further delay aging (Padilha et al., 2021).

Under normal physiological conditions, the aerobic metabolism of organisms continuously produces oxygen free radicals (ROS) which cause damage to cells. When the burden of damage caused by these ROS is greater than the repair capacity of the body, bodily aging results (Liu et al., 2022). During the aging process, the activities of antioxidant enzymes are significantly reduced. This causes the continuous accumulation of free radicals in the body, which consequently affects normal metabolism, increases bodily fatigue, and reduces the capacity for exercise, thereby ultimately resulting in the decline of the quality of life for the elderly (Tan et al., 2000). This trajectory illustrates a close relationship between oxidative stress, aging, and exercise capacity. Enhancing exercise function by bolstering the antioxidant processes within the body represents an effective way to delay aging. Slowing the process of aging, in turn, strengthens the vitality of the organs and muscles, thus promoting athletic function. This strategy comprises one effective method which can be used to delay physical fatigue; namely, by supplementing exogenous antioxidants to the body, thereby preventing the oxidation of easily-oxidized substrates in cells, inhibiting lipid peroxidation, and directly eliminating oxygen free radicals to reduce oxidative stress in the body (Kerksick and Willoughby, 2005; McLeay et al., 2017).

The development and utilization of probiotic lactic acid bacteria (LAB) comprises an active area of research in the field of food bioengineering. Numerous studies have been conducted on the prebiotic function of LAB to show that many employ antioxidant functions, though there exist differences in these functions between different strains. Zhang et al. (2021) Found that *Lactobacillus fermentum* HFY03 isolated from yak yogurt could significantly improve the running duration of mice, reduce their contents of urea nitrogen and lactic acid, and increase their levels of catalase and superoxide dismutase in the liver tissue, thereby indicating that *L. fermentum* HFY03 exerts an antifatigue effect through improving the antioxidant capacity of mice that run until exhaustion. Some studies have also found that the use of LAB that employ antioxidant functions could improve the antioxidant properties of fermentation-based products. Zhou et al. (2021) used *Lactobacillus plantarum* HFY09 to ferment soymilk and found that the resulting fermented soymilk could effectively alleviate the accelerated aging of mice induced by D-galactose (Zhou et al., 2021). There are many studies existing on probiotics, but at present, there are few reports existing on their impacts on exercise capacity. In this study, a new lactic acid bacterium, *Lactobacillus plantarum* KSFY01 (*L. plantarum* KSFY01), was isolated from traditional fermented yak yogurt. It possessed high resistance to the conditions of pH 3.0, artificial gastric juice, and 0.3% bile salt. The results showed that *L. plantarum* KSFY01 was mostly unaffected by artificial gastric juice, and its survival rate was 86.26%. The growth rate in 0.3% bile salt was 54.24%. This study then constructed a mouse model of accelerated aging by the injection of D-galactose injection to investigate the effect of *L. plantarum* KSFY01 on the antioxidant levels and anti-fatigue response of mice. The results are intended to provide a reference for the application of *L. plantarum* in functional foods and for the development of health products.

## 2. Materials and methods

### 2.1. Experimental microorganism strain

This study isolated a *L. plantarum* strain from yak yogurt obtained from Kashgar, Xinjiang, and named it *L. plantarum* KSFY01. The bacteria were stored frozen at-80°C and subsequently inoculated into sterilized MRS medium for resuscitation. The resuscitation conditions involved 37°C, culturing for 18–24 h, and the bacterial activation spanning two generations. The second generation of bacterial suspension was then taken and centrifuged at 5000 rpm/min for 10 min. The upper culture medium was discarded, while that which remained was added with the same volume of 0.9% physiological saline to make up the bacterial suspension. Using the gradient dilution method, the *L. plantarum* KSFY01 bacterial solution was diluted to 1.0 × 10^10^ and 1.0 × 10^9^ colony-forming units (CFU).

### 2.2. Animal experiment

**Figure 1:**
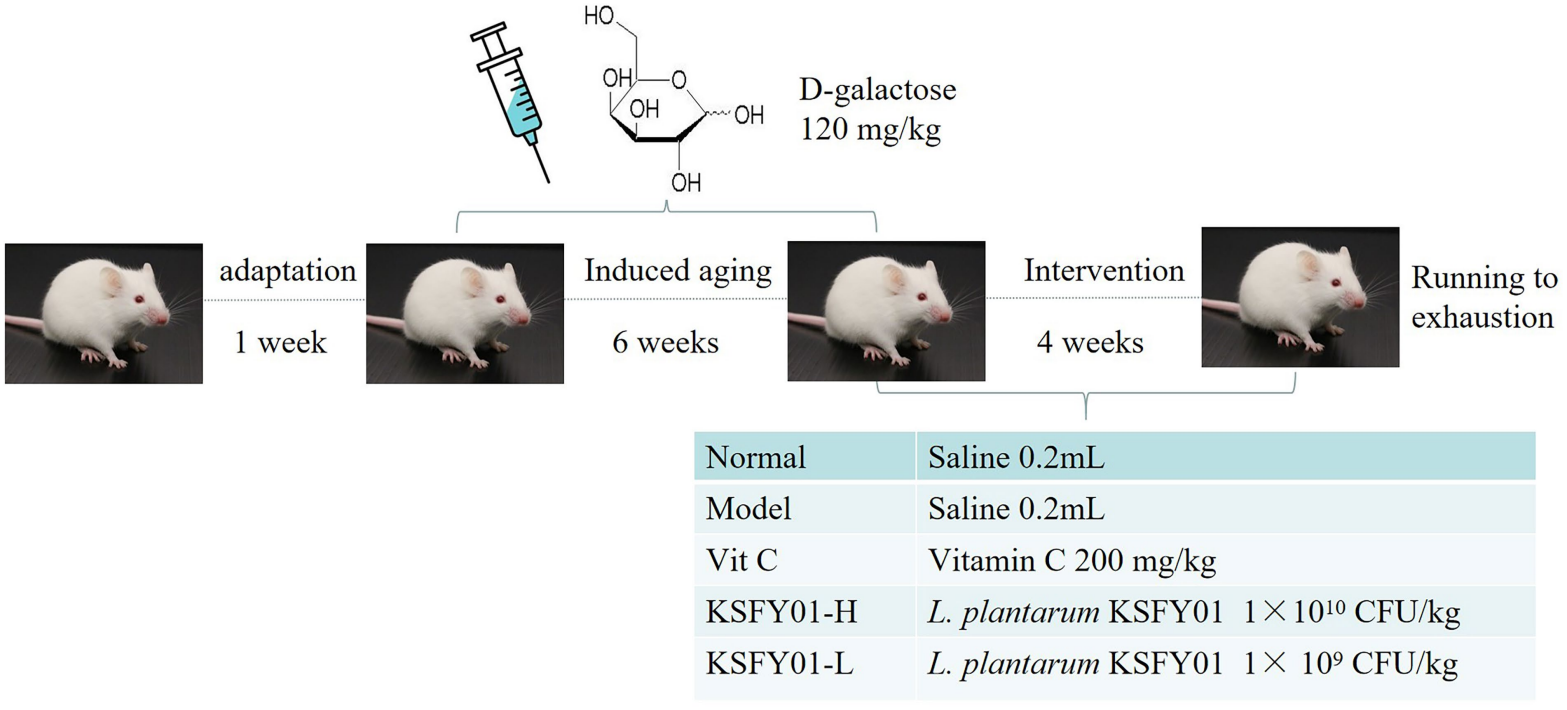
Experimental design.

Fifty 6-week-old male Kunming mice were purchased from the animal experiment center of Chongqing Medical University. Before the formal experiment commenced, the mice were placed under a 12-h light/dark cycle for 1 week of adaptive feeding with free access to food and water. At the end of the one-week adaptation period, the mice were equally divided into 5 groups (with 10 mice each): the normal group, model group, vitamin C group (Vit C), KSFY01 high-dose group (KSFY01-H), and KSFY01 low-dose group (KSFY01-L). The duration of the experimental period was 10 weeks (Figure 1). After the start of the experiment, the mice in all groups except the normal group were injected with D-galactose solution (120 mg/kg) intraperitoneally every day for 6 weeks, while the mice in the normal group were intraperitoneally injected with the same amount of saline. From the 7th week onwards, the mice in the normal group and the model group were gavaged with distilled water (0.02 ml/kg) every day; the mice in the Vit C group were gavaged with 200 mg/kg of vitamin C every day; the mice in the KSFY01-H group were gavaged every day with *L. plantarum* KSFY01 bacterial suspension at a dose of 1.0 × 10^10^ CFU/kg; mice in the KSFY01-L group were administered the *L. plantarum* KSFY01 bacterial suspension at a dose of 1.0 × 10^9^ CFU/kg for 4 weeks. After 10 weeks, the mice were subjected to the running exhaustion test, whereby the time to exhaustion of each group of mice was recorded. Then, the mice were immediately sacrificed, had their blood was sampled from the eyeballs, and their liver and kidney tissues were promptly collected for subsequent experiments.

### 2.3. Running test

After 10 weeks, the mice were subjected to the running exhaustion test. The running wheel was set to 20 rpm/min, and the mice were forced to run on the wheel (YH-CS, Wuhan Yihong Technology Co., Ltd., Wuhan, Hubei, China). When the mice stopped running, electric shocks were performed 5 consecutive times until the mice did not run, thereby indicating exhaustion, and the running time was recorded.

### 2.4. Biochemical and energy metabolism indices

Mice eyeball blood was collected into 1.5 ml centrifuge tubes, placed in a 4°C refrigerator for 30 min, and then centrifuged (3,000 rpm at 4°C for 10 min). An appropriate amount of serum was then taken and analyzed with biochemical kits (Nanjing Jiancheng Bioengineering Institute, Nanjing, China) to determine the contents of blood urea nitrogen (BUN), serum free fatty acid (NEFA), lactic acid (LA), lactate dehydrogenase (LDH), catalase (CAT), glutathione (GSH), malondialdehyde (MDA) levels, and muscle glycogen (MG). The enzyme-linked immunosorbent assay (ELISA; Shanghai Enzyme Link Biotechnology Co., Ltd., Shanghai, China) was used to determine the activities of aspartate aminotransferase (AST), alanine aminotransferase (ALT), and creatine kinase (CK) in the mouse serum.

### 2.5. Histopathological analysis

Immediately after the mice were dissected, portions of the mouse liver and kidney tissues were taken and placed in a 10% neutral formalin solution for fixation. The tissue samples were embedded, cut into 5–10 μm slices, and then stained with hematoxylin–eosin (H&E) dye (Li et al., 2021a). The pathological changes in the tissue were then observed under a light microscope.

### 2.6. Real-time quantitative PCR detection

The mRNA expression of SOD1, SOD2, CAT, interleukin-1β (IL-1β), tumor necrosis factor α (TNF-α), interleukin-10 (IL-10), heme oxygenase-1 (HO-1), nuclear factor-erythroid 2 related factor 2 (Nrf2), γ-glutamylcysteine synthetase (γ-GCS), and NAD(P)H dehydrogenase [quinone] 1 (NQO-1) were measured using real-time quantitative PCR (RT-qPCR; Wang et al., 2021). The extraction of mouse RNA from the liver and muscle tissue was conducted as follows. Mouse liver tissue and muscle tissue were removed from storage at −80°C, and 100 mg of each was then weighed into a homogenization tube, added with 1 ml of TRIzol reagent, and homogenized using beads for extraction. A nucleic acid analyzer was then used to measure the concentration of the extracted RNA to ensure that the purity value was between 1.8 and 2.0. The RNA was then reverse-transcribed into cDNA using a kit (Thermo Fisher Scientific, Inc., Waltham, MA, United States) according to the manufacturer’s instructions, after which the resulting cDNA was aliquotted and stored at −80°C for later use. The reverse-transcribed cDNA was then used as a template whereby it was added with fluorescent dye and placed into a fluorescence quantitative PCR instrument to carry out the reaction. The volume used for the RT-PCR reaction system was 20 μl, and the reaction conditions were as follows: preheating at 95°C for 60 s, 40 cycles of 95°C for 15 s, 55°C for 30 s, 72°C for 35 s, and then ultimately tested at 95°C for 30 s and 55°C for 35 s. Each reaction was performed four times (Long et al., 2020). In the experiment, the β-actin gene was used as the internal reference gene, and the relative expression level of the target mRNA was determined by the 2^−ΔΔCt^ relative quantitative method (Li et al., 2021b). The respective primer sequences of the internal reference gene and target gene used in this study are shown in Table 1.

**Table 1:** Primer sequences used in the real-time quantitative PCR.

Gene names	Primer sequence
SOD1	Forward: 5′-AACCAGTTGTGTTGTCAGGAC-3′
	Reverse: 5′-CCACCATGTTTCTTAGAGTGAGG-3′
SOD2	Forward: 5′-CAGACCTGCCTTACGACTATGG-3′
	Reverse: 5′-CTCGGTGGCGTTGAGATTGTT-3′
CAT	Forward: 5′- GGAGGCGGGAACCCAATAG-3′
	Reverse: 5′-GTGTGCCATCTCGTCAGTGAA-3′
IL-1β	Forward: 5′-GAAATGCCACCTTTTGACAGTG-3′
	Reverse: 5′-TGGATGCTCTCATCAGGACAG-3′
IL-10	Forward: 5′-CTTACTGACTGGCATGAGGATCA-3′
	Reverse: 5′-GCAGCTCTAGGAGCATGTGG-3′
TNF-α	Forward: 5′-CAGGCGGTGCCTATGTCTC-3v
	Reverse: 5′-GCTGCAACAGGGGGTAACAT-3′
Nrf2	Forward: 5′-CAGTGCTCCTATGCGTGAA-3*v*
	Reverse: 5′-GCGGCTTGAATGTTTGTC-3′
HO-1	Forward: 5′-ACAGATGGCGTCACTTCG-3′
	Reverse: 5′-TGAGGACCCACTGGAGGA-3′
γ-GCS	Forward: 5′-GCACATCTACCACGCAGTCA-3′
	Reverse: 5′-CAGAGTCTCAAGAACATCGCC-3′
NQO1	Forward: 5′-CTTTAGGGTCGTCTTGGC-3′
	Reverse: 5′-CAATCAGGGCTCTTCTCG-3′
β-actin	Forward: 5′-CATGTACGTTGCTATCCAGGC-3′
	Reverse: 5′-CTCCTTAATGTCACGCACGAT-3′

### 2.7. Statistical analysis

All data are expressed as the mean ± standard deviation and plotted using GraphPad Prism (version 7.00) software. SPSS software (SPSS 22.0, SPSS Inc.) was used for statistical analysis of the data, and ANOVA and Duncan’s test were used to evaluate the significance of the differences for each parameter of the samples (*p* < 0.05).

## 3. Results

### 3.1. Effects of *Lactobacillus plantarum* KSFY01 on exercise endurance In accelerated-aging mice

**Figure 2:**
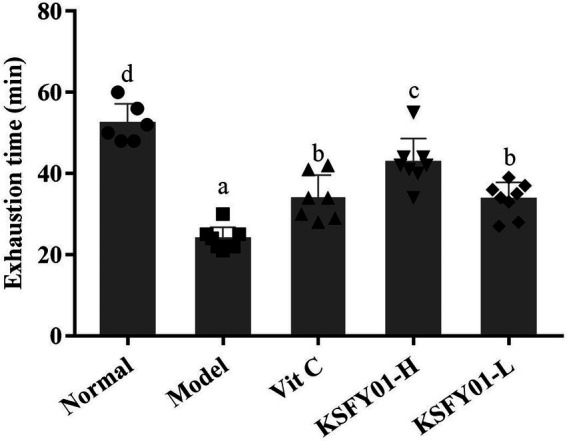
Running exhaustion time of mouse in each group. ^a–d^Values in the same column with different letter superscripts indicate significant difference (*p* < 0.05). Vit C, mice treated with vitamin C (200 mg/kg); KSFY01-H, mice treated with *L. plantarum* KSFY01 (1 × 10^10^ CFU/kg); KSFY01-L, mice treated with *L. plantarum* KSFY01 (1 × 10^9^ CFU/kg).

It can be seen from Figure 2 that the running time of the mice in the normal group was the greatest among all the groups, while the running time in the aging model group was the shortest. Compared with the aging model group, both *L. plantarum* KSFY01 and vitamin C could significantly (*p* < 0.05) prolong the running time of aging mice, and the *L. plantarum* KSFY01 high-dose group had the greatest prolongation effect, which was significantly better than that of either the low-dose *L. plantarum* KSFY01 group or the Vit C group. These experimental results show that *L. plantarum* KSFY01 can improve the exercise endurance of mice, prolong the exercise time of mice, and relieve their exercise-induced fatigue.

### 3.2. Energy metabolism in mice

As shown in Table 2, the serum levels of BUN, NEFA, and LD in the normal group were significantly (*p* < 0.05) lower than those in other groups, while the LDH and MG levels were significantly (*p* < 0.05) higher than those in other groups. Conversely, the above indicators in the serum of mice in the aging model group showed the opposite trend to that of the normal group. Compared with the aging model group, *L. plantarum* KSFY01 and vitamin C each reduced the levels of BUN, NEFA, and LD in the serum of aging mice, while having increased the levels of LDH and MG. These experimental results show that *L. plantarum* KSFY01 can reduce the BUN produced by mice following exercise, increase the activity of LDH, reduce the content of LD, and relieve the exercise-induced fatigue of mice.

**Table 2:** The MG, BUN, NEFA, LD, and LDH levels in aging mouse.

Group	MG (mg/mL)	BUN (mmol/L)	NEFA (μmol/L)	LD (mmol/L)	LDH (U/L)
Normal	5.00 ± 0.17^c^	3.27 ± 0.69^a^	443.78 ± 32.89^a^	2.12 ± 0.07^a^	325.22 ± 24.94^c^
Model	1.27 ± 0.17^a^	7.23 ± 0.50^c^	1459.14 ± 82.26^e^	6.80 ± 1.00^d^	195.43 ± 26.42^a^
Vit C	2.67 ± 0.15^b^	3.46 ± 0.81^a^	853.55 ± 134.45^c^	3.52 ± 0.62^ab^	308.07 ± 68.53^c^
KSFY01-H	2.84 ± 0.17^b^	3.50 ± 0.79^a^	710.75 ± 52.32^b^	3.74 ± 0.38^b^	298.88 ± 17.74^bc^
KSFY01-L	2.66 ± 0.32^b^	5.64 ± 0.71^b^	1071.61 ± 71.53^d^	5.00 ± 0.77^c^	252.17 ± 70.00^b^

The data are expressed as the mean ± SD. ^a–d^Values in the same column with different letter superscripts indicate significant difference (*p* < 0.05). Vit C, mice treated with vitamin C (200 mg/kg); KSFY01-H, mice treated with *L. plantarum* KSFY01 (1 × 10^10^ CFU/kg); KSFY01-L, mice treated with *L. plantarum* KSFY01 (1 × 10^9^ CFU/kg).

### 3.3. Sports injuries in mice

It can be seen from Table 3 that the ALT, AST, and CK contents of the vitamin C group, *L. plantarum* KSFY01 high dose group, and *L. plantarum* KSFY01 low dose groups lay between the results of the model group and the normal group. Both the *L. plantarum* KSFY01 bacterial suspension and vitamin C had good alleviating effects by decreasing the high ALT, AST, and CK contents induced by D-galactose. The effects of the high-dose *L. plantarum* KSFY01 group and the vitamin C group were better than that of the low-dose *L. plantarum* KSFY01 group.

**Table 3:** The ALT, AST, and CK levels in aging mouse.

Group	ALT (ng/mL)	AST (ng/mL)	CK (ng/mL)
Normal	9.15 ± 0.62^a^	17.24 ± 1.42^a^	61.89 ± 5.65^a^
Model	19.78 ± 1.77^d^	39.83 ± 0.96^e^	120.34 ± 3.25^d^
Vit C	11.30 ± 1.66^b^	24.52 ± 0.41^c^	77.67 ± 8.45^b^
KSFY01-H	10.87 ± 1.81^b^	19.91 ± 1.36^b^	72.26 ± 6.27^b^
KSFY01-L	16.32 ± 0.75^c^	30.51 ± 0.89^d^	95.18 ± 3.95^c^

The data are expressed as the mean ± SD. ^a–d^Values in the same column with different letter superscripts indicate significant difference (*p* < 0.05). Vit C, mice treated with vitamin C (200 mg/kg); KSFY01-H, mice treated with *L. plantarum* KSFY01 (1 × 10^10^ CFU/kg); KSFY01-L, mice treated with *L. plantarum* KSFY01 (1 × 10^9^ CFU/kg).

### 3.4. Serum oxidation level of mice

The serum levels of CAT, GSH, and MDA were compared between groups, as shown in Table 4. Compared with the normal group, CAT enzyme activity in the model group was significantly decreased (*p* < 0.05), and the GSH content was also significantly decreased. After either vitamin C or *L. plantarum* KSFY01 intervention, the CAT enzyme activity and GSH level were both increased; among these, the high dose of *L. plantarum* KSFY01 was more effective than either vitamin C or the low dose of *L. plantarum* KSFY01. Compared with the model group, vitamin C and *L. plantarum* KSFY01 significantly reduced the MDA content (*p* < 0.05); among these, high-dose *L. plantarum* KSFY01 had the greatest effect.

**Table 4:** The CAT, GSH, and MDA levels in aging mouse.

Group	CAT (U/mL)	GSH (μmol/L)	MDA (nmol/mL)
Normal	11.34 ± 2.10^b^	78.07 ± 8.37^d^	4.36 ± 0.53^a^
Model	5.45 ± 1.71^a^	42.52 ± 8.88^a^	15.68 ± 2.00^e^
Vit C	7.27 ± 0.99^a^	65.78 ± 12.06^c^	9.73 ± 1.03^c^
KSFY01-H	10.15 ± 0.75^b^	72.87 ± 3.18^cd^	6.71 ± 1.16^b^
KSFY01-L	6.07 ± 1.39^a^	55.04 ± 10.15^b^	12.70 ± 1.57^d^

The data are expressed as the mean ± SD. ^a–d^Values in the same column with different letter superscripts indicate significant difference (*p* < 0.05). Vit C, mice treated with vitamin C (200 mg/kg); KSFY01-H, mice treated with *L. plantarum* KSFY01 (1 × 10^10^ CFU/kg); KSFY01-L, mice treated with *L. plantarum* KSFY01 (1 × 10^9^ CFU/kg).

### 3.5. Pathological evaluation

**Figure 3:**
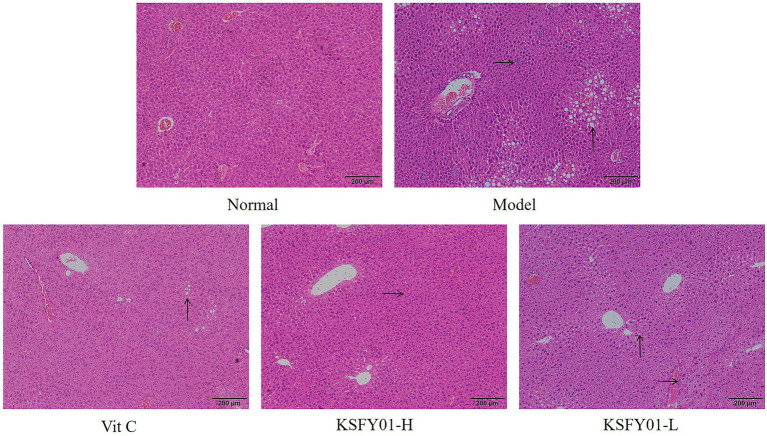
H&E pathological observation of liver tissue in aging mouse (magnification, ×100). Vit C, mice treated with vitamin C (200 mg/kg); KSFY01-H, mice treated with *L. plantarum* KSFY01 (1 × 10^10^ CFU/kg); KSFY01-L, mice treated with *L. plantarum* KSFY01 (1 × 10^9^ CFU/kg). → indicate adipocyte; ↑ indicate inflammatory cell in liver.

The morphology of liver tissue under the microscope in mice is shown in Figure 3. The liver lobules of the mice in the normal group exhibited a clear structure and no obvious changes in the intact liver cells, and there were no phenomena such as granular degeneration, vacuolar degeneration, hepatocyte enlargement, or necrosis. In the D-galactose-induced aging model group, the structure of the hepatic lobules of the mice was destroyed, the arrangement of hepatocytes was disordered, many vacuoles of different sizes appeared in the encapsulation, and there was inflammatory infiltration accompanied by cell necrosis. Both vitamin C and *L. plantarum* KSFY01 could reduce hepatocyte damage in aging mice. After the action of KSFY01-H, the structure of hepatic lobules was mainly intact, and the degeneration of hepatocytes was significantly improved. There were still vacuoles of different sizes in the liver tissue of mice observed in both the Vit C group and KSFY01-L group.

**Figure 4:**
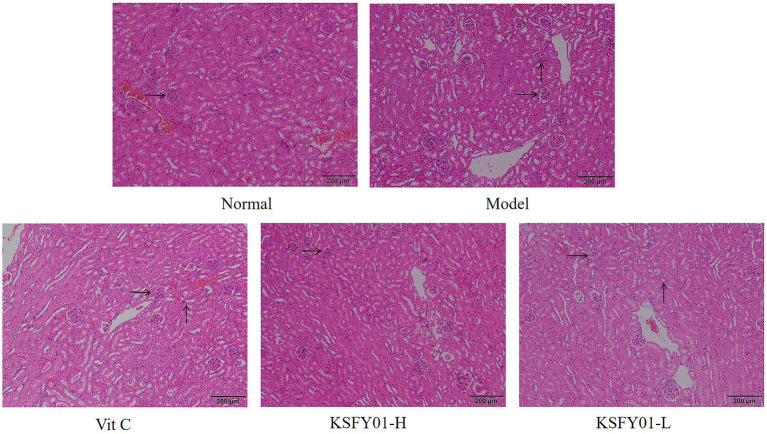
H&E pathological observation of kidney tissue in aging mouse (magnification ×100). Vit C, mice treated with vitamin C (200 mg/kg); KSFY01-H, mice treated with *L. plantarum* KSFY01 (1 × 10^10^ CFU/kg); KSFY01-L, mice treated with *L. plantarum* KSFY01 (1 × 10^9^ CFU/kg). → indicate glomerulus; ↑ indicate inflammatory cell in kidney.

The morphology of kidney tissue under the microscope of mice is shown in Figure 4, and the glomerular vascular loops in the normal group were thin and clear. The number of endothelial and mesangial cells was normal. The surrounding renal tubules were also normal. The glomeruli in the kidney tissue of the mice in the aging model group were irregular in shape, some were ruptured or swollen, and there was inflammatory cell infiltration observed between the tissues. Both vitamin C and *L. plantarum* KSFY01 could reduce kidney tissue damage caused by aging, and the infiltration of inflammatory cells decreased to varying degrees.

### 3.6. Expression of mRNA in liver tissue

**Figure 5:**
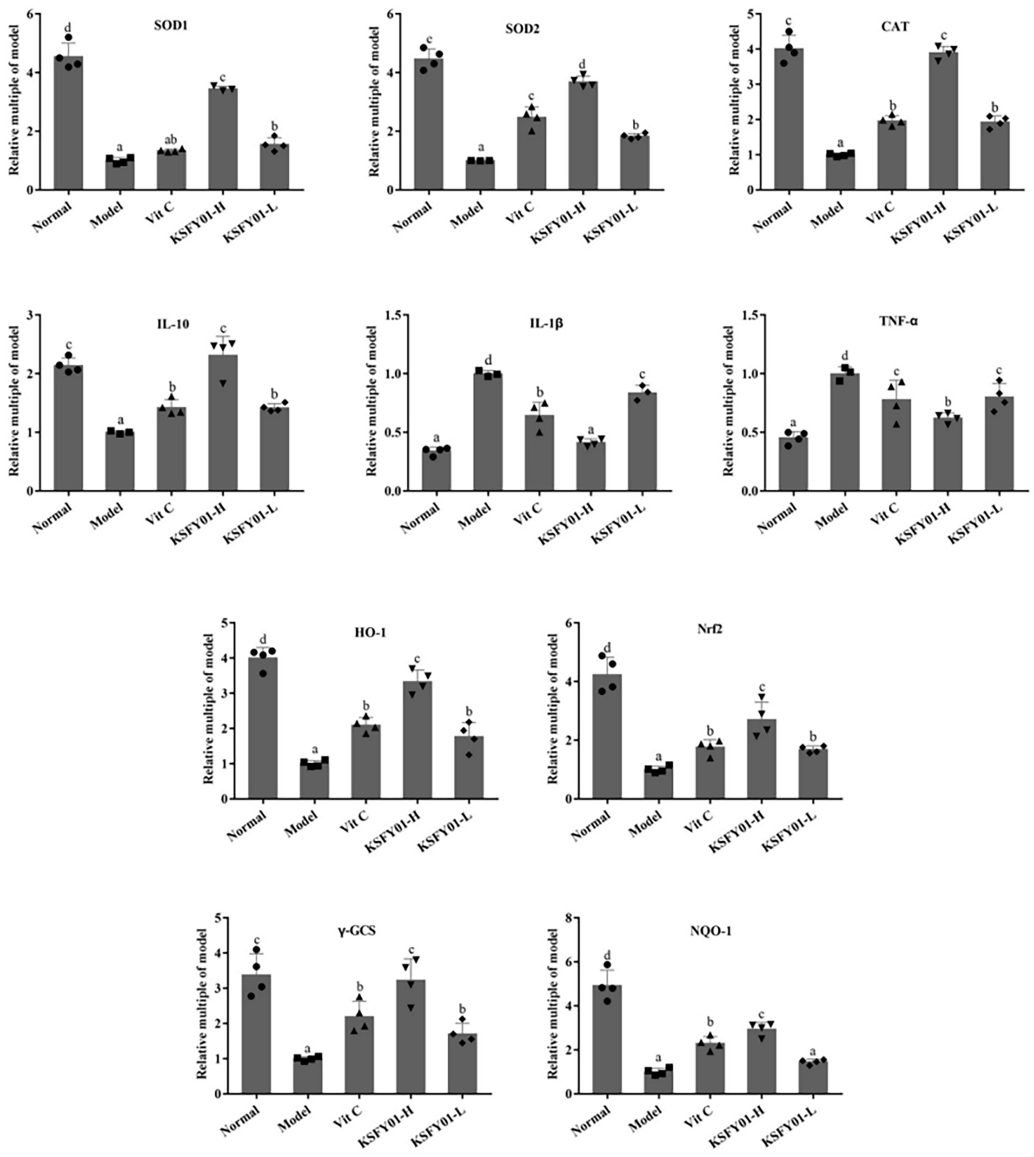
The SOD1, SOD2, CAT, IL-1β, IL-10, TNF-α, HO-1, Nrf2, γ-GCS, and NQO-1 mRNA expressions in mouse liver tissue. ^a–e^Values in the same column with different letter superscripts indicate significant difference (*p* < 0.05). Vit C, mice treated with vitamin C (200 mg/kg); KSFY01-H, mice treated with *L. plantarum* KSFY01 (1 × 10^10^ CFU/kg); KSFY01-L, mice treated with *L. plantarum* KSFY01 (1 × 10^9^ CFU/kg).

As shown in Figure 5, the expression of IL-1β and TNF-α mRNA in the liver tissue of the normal mice was the lowest, while the expression of IL-10, SOD1, SOD2, CAT, HO-1, Nrf2, γ-GCS, and NQO1 were the greatest. The model group showed the opposite trend. Vitamin C and *L. plantarum* KSFY01 could each downregulate IL-1β and TNF-α expression while upregulating IL-10, SOD1, SOD2, CAT, HO-1, Nrf2, γ-GCS, and NQO1 expression. The high-dose *L. plantarum* KSFY01 group had the strongest ability to cause these expression changes and gave the most similar values to those of the normal mice.

### 3.7. Expression of mRNA in mouse skeletal muscle tissue

**Figure 6:**
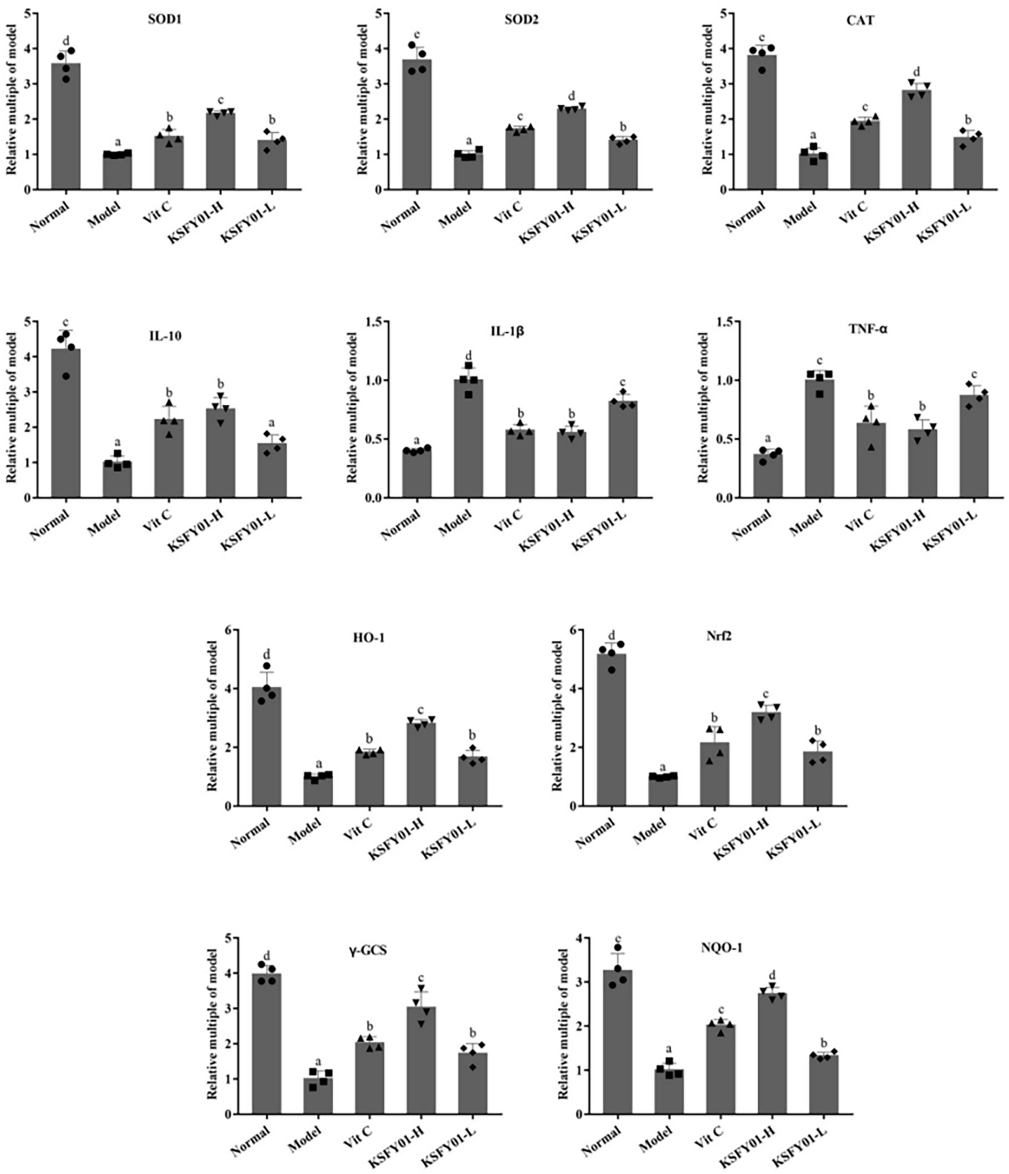
The SOD1, SOD2, CAT, IL-1β, IL-10, TNF-α, HO-1, Nrf2, γ-GCS, and NQO-1 mRNA expressions in mouse skeletal muscle tissue. ^a–e^Values in the same column with different letter superscripts indicate significant difference (*p* < 0.05). Vit C, mice treated with vitamin C (200 mg/kg); KSFY01-H, mice treated with *L. plantarum* KSFY01 (1 × 10^10^ CFU/kg); KSFY01-L, mice treated with *L. plantarum* KSFY01 (1 × 10^9^ CFU/kg).

We also analyzed Nrf2 pathway-related genes in mouse muscle tissue. IL-10, IL-1β, TNF-α, SOD1, SOD2, CAT, HO-1, Nrf2, γ-GCS, and NQO1 are oxidative genes involved in this pathway. These genes become abnormally expressed when the expression of Nrf2 changes. As shown in Figure 6, compared with the normal group, the expression levels of IL-10, Cu/Zn-SOD, Mn-SOD, CAT, HO-1, Nrf2, γ-GCS, and NQO1 mRNA in the muscle tissue of the model group were significantly decreased (*p* < 0.05). Conversely, the expression of IL-1β and TNF-α mRNA increased significantly (*p* < 0.05). Compared with the model group, the vitamin C and *L. plantarum* KSFY01 interventions each greatly improved the expression of these genes (*p* < 0.05) to the degree that they more resembled the normal group. The regulatory effect of *L. plantarum* KSFY01 was enhanced in a dose-dependent manner. The regulatory effect of high dose *L. plantarum* KSFY01 was greater than that of low dose *L. plantarum* KSFY01. These results indicate that *L. plantarum* KSFY01 has a positive effect on aging-induced muscle damage in mice.

## 4. Discussion

With increasing age, the generation of endogenous free radicals increases in parallel, while homeostasis, stress resilience, and the function of the endogenous antioxidant system all gradually decline. With the abnormal and excessive accumulation of free radicals in the body, the balance between the oxidation and antioxidant systems in the body breaks down, thereby damaging organelles such as the mitochondria and cell membranes. These changes result in cellular dysfunction, in turn affecting the metabolism and accelerating the aging process (Qian et al., 2018). As people age, the conduction velocity of their motor and sensory nerves gradually decreases, and this decline in the function of the nervous system causes the weakening of motor ability, which is accompanied by the frequent occurrence of fatigue (Wu et al., 2022). Exercise can help clear free radicals and thereby allow normal cells to maintain the integrity of mitochondrial structure and function; simultaneously, being in good physical condition can facilitate the body to maintain exercise capacity (Vargas-Mendoza et al., 2021). Improvements in exercise capacity represent the most direct manifestation of bodily resistance to fatigue, and also serve as a key manifestation of resistance to aging (Zhong et al., 2022). Studies have confirmed that some LAB possess good antioxidant and anti-aging effects (Suo et al., 2018). This study also established a mouse aging model by the intraperitoneal injection of D-galactose. This was done to observe the effect of *L. plantarum* KSFY01 on endurance running in mice in an attempt to verify the intervention effect of *L. plantarum* KSFY01 on exercise capacity in the aging state. The experimental results also confirmed that *L. plantarum* KSFY01 could prolong the endurance running time of mice, enhance their exercise capacity, and relieve exercise-induced fatigue.

Long-term continuous exercise causes a substantial degree of bodily fatigue. In this study, we designed a high-intensity exercise treadmill experiment to induce exercise fatigue in mice. The depletion of hepatic glycogen and the accumulation of metabolic byproducts are each thought to contribute to bodily fatigue (Wang et al., 2015; Yi et al., 2021; Zhu et al., 2021). Physical exercise initiates with increases in aerobic muscle activity. If the exercise is intense, anaerobic metabolism will be employed, thereby resulting in the accumulation of LA. Increasing LA then reduces the pH value of both tissues and blood, consequently affecting the physiological and biochemical processes occurring in the body, and ultimately affecting bodily function (Peng et al., 2021). In this way, LA is an important indicator for measuring the level of fatigue. The normal function of LDH in cells is to catalyze the interconversion between pyruvate and lactate (Majid, 2022). BUN is a by-product of metabolic proteins that is produced by the body after excessive glycogen consumption, and it represents a sensitive parameter related to fatigue (Yang et al., 2022). The lower the physical endurance of an individual, the greater the increase in BUN levels during physical exertion (Lee et al., 2021). Therefore, serum levels of LA, LDH, and BUN can indicate both the speed and extent of fatigue that has developed within the body. In addition, the physical exhaustion of mice during long-term high-intensity exercise is related to changes in MG content. After a period of exercise, glycogen in the body is decomposed into lactic acid, and energy is then released to allow muscle activity. The greater the MG reserve, the better the endurance of the body, and the longer the exercise duration (Kim et al., 2020). In this study, compared with the model group, vitamin C and *L. plantarum* KSFY01 interventions each significantly attenuated the accumulation of these metabolites and regulated blood sugar levels. *L. plantarum* KSFY01 also reduced muscle damage by regulating LDH levels. This indicates that *L. plantarum* KSFY01 can relieve fatigue by effectively reducing the accumulation of metabolic byproducts, improving muscle damage, and enhancing energy storage capacity.

There is increasing evidence that high-intensity and acute exercise can each cause damage to hepatocytes by reducing blood flow to the liver and portal vein, which often leads to hepatocyte hypoxia, and ultimately, liver necrosis (Kinoshita et al., 2003). This injury is often accompanied by elevated levels of CK, AST, and ALT (Sangita et al., 2018). In this study, we also found that exercise fatigue caused a significant increase in the liver function indicators of serum CK, ALT, and AST, thereby indicating liver dysfunction. This outcome could be reversed by either the oral administration of vitamin C or *L. plantarum* KSFY01.

The liver is the central organ of oxidative stress reaction, which can significantly reflect the degree of oxidation of the body, and is also an organ easy to produce free radicals and lipid peroxides. The increase of free radicals caused by aging can be clearly shown in the pathological changes of liver tissue. In addition, with the aging of the body, the kidney gradually shrinks, and the secretion of prostaglandins decreases, leading to vasoconstriction and reduced blood flow. As aging continues, the vascular contraction frequency of liver and kidney decreases, and the blood supply of various organs decreases, which directly reduces the exercise ability (Aschner et al., 2021). In this study, pathological section observation also confirmed that aging led to liver and kidney tissue damage, *L. plantarum* KSFY01 can effectively reduce the decline and damage of liver and kidney tissues, and one of the important roles it plays may be to improve motor function.

During exercise, many free radicals (such as hydroxyl radicals and superoxide anion radicals) are generated, and this phenomenon is especially pronounced in the aged state (Mariusz and Sławomir, 2013). CAT, GSH, etc. are all important antioxidants and free radical scavengers in the human body. Their existence can maintain the normal oxidative stress homeostasis by removing excess free radicals. The excessive accumulation of ROS can cause oxidative stress and attack biological macromolecules, such as lipids, proteins, and nucleic acids, to form lipid peroxidation products, like MDA (Kheradmand et al., 2009; Li et al., 2022). MDA further damages the structure of the cell membrane, resulting in cell swelling and necrosis. After the intervention of either vitamin C or *L. plantarum* KSFY01, the content of MDA in the blood of mice was decreased, while the contents of CAT and GSH were increased. Therefore, our results suggest that *L. plantarum* KSFY01 has potent antioxidant activity and can be used to inhibit aging, whereby it exerts the effect of enhancing exercise endurance and performance.

In terms of inflammatory response, with the growth of age, the function of the immune system gradually declines, which induces some diseases that seriously affect tissues and organs, and aggravates the aging of various systems of the body. TNF-α and IL-1β can activate T cells and enhance the immune response of the body. The results showed that in the model group, proinflammatory factor levels (TNF-α and IL-1β) were the highest, while anti-inflammatory factor levels (IL-10) were the lowest. In the normal group, a completely opposite trend was noted. After treatment with KSFY01, the expression of TNF-α and IL-1β was inhibited, while the expression of IL-10 was enhanced, indicating that the *L. plantarum* KSFY01 inhibits the production of inflammatory factors.

To elucidate the molecular mechanism by which *L. plantarum* KSFY01 affects fatigue and fatigue-related organ dysfunction in the aging state, we measured the expression of oxidative stress-related signaling genes in the liver and muscle tissue of mice. Many studies have shown that the Nrf2 signaling pathway serves as an extremely important endogenous defense system in the body (Sunil et al., 2021). Nrf2 is the most critical in the response to oxidative stress in cell. The protein primarily interacts with antioxidant response elements (AREs) to induce the expression of encoded antioxidant proteins and phase II detoxification enzymes (Johnson et al., 2008), thereby increasing cellular resistance against harmful stimuli within cells to play an important protective role. This confers anti-tumor, anti-stress, anti-apoptotic, anti-inflammatory, and neuroprotective effects (Vriend and Reiter, 2015; Zhang et al., 2022). Downstream antioxidant enzymes that are regulated by the Nrf2 signaling pathway include γ-GCS, SOD, CAT, and CSH-Px (Na and Surh, 2005), while the downstream phase II detoxification enzymes that are regulated by the Nrf2 signaling pathway include HO-1 and NQO1 (Keum, 2012). Oxidative stress generates a large amount of ROS to cause peroxidative damage, while the increase of ROS in mitochondria can lead to progressive lipid oxidation, generate more lipid peroxides, and induce tumor necrosis factor TNF-α (Wang W. et al., 2022; Wang L. et al., 2022). TNF-α not only damages the function of the mitochondrial respiratory chain and affects the electron transfer in the respiratory chain, but also opens the permeability transition pore of mitochondria, depletes cytochrome C in mitochondria, and then triggers the apoptosis and necrosis of liver cells (Wang W. et al., 2022; Wang L. et al., 2022). In this study, we used RT-PCR to analyze whether vitamin C and *L. plantarum* KSFY01 could affect the expression of these genes. The results showed that high-intensity exercise decreased the mRNA expression levels of Nrf2, NQO1, γ-GCS HO-1, SOD1, SOD2, CAT, and IL-10 in both liver and muscle tissue. Conversely, vitamin C and *L. plantarum* KSFY01 significantly increased the expression of these genes, and the intervention of *L. plantarum* KSFY01 was more significant. The intervention effect of *L. plantarum* KSFY01 was dose-dependent, and the intervention effect of high-dose *L. plantarum* KSFY01FDB was better than that of low-dose *L. plantarum* KSFY01. These findings suggest that the Nrf2 pathway is the underlying molecular mechanism by which *L. plantarum* KSFY01 exerts its antioxidant and anti-fatigue effects.

## 5. Conclusion

In this study, we established a mouse aging model to explore the antioxidant effect of *L. plantarum* KSFY01 and its effect on improving the ability of mice to run. The experimental results show that *L. plantarum* KSFY01 could alleviate exercise-induced fatigue and improve the exercise capacity of aging mice by improving their metabolite accumulation, glycogen storage, muscle and liver damage, and levels of oxidative stress. These results provide a reference for the future development of food-derived antioxidants for anti-fatigue effects, and for improving the motor function of the elderly. While these findings are promising, this study only examined the effect of LP-KFY04 in an animal model, so further experimentation is required to determine if a similar effect can be achieved in humans. In addition to fatigue, many diseases are related to oxidative stress, such as aging, diabetes and nonalcoholic fatty liver. In future research, other effects of *L. plantarum* KSFY01 can be further explored.

## Data availability statement

The raw data supporting the conclusions of this article will be made available by the authors, without undue reservation.

## Ethics statement

The animal study was reviewed and approved by the Ethics Committee of Chongqing Collaborative Innovation Center for Functional Food.

## Author contributions

QC performed the majority of the experiments and wrote the manuscript. CL, YZ, and SW contributed to the data analysis. FL designed and supervised the study and checked the final manuscript. All authors contributed to the article and approved the submitted version.

## Funding

This research was funded by the Science and Technology Project of Chongqing (cstc2021jcyj-msxmX0408), the Science and Technology Project of Chongqing Education Commission (KJQN202001614).

## Conflict of interest

The authors declare that the research was conducted in the absence of any commercial or financial relationships that could be construed as a potential conflict of interest.

## Publisher’s note

All claims expressed in this article are solely those of the authors and do not necessarily represent those of their affiliated organizations, or those of the publisher, the editors and the reviewers. Any product that may be evaluated in this article, or claim that may be made by its manufacturer, is not guaranteed or endorsed by the publisher.
